# Abundant Intra-Subtype Reassortment Revealed in H13N8 Influenza Viruses

**DOI:** 10.3390/v16040568

**Published:** 2024-04-07

**Authors:** Sofia Feoktistova, Marya Sayganova, Kseniya Trutneva, Olga Glazova, Artem S. Blagodatski, Liudmila Shevkova, Anna Navoikova, Yuriy Anisimov, Eugene Albert, Olga Mityaeva, Pavel Volchkov, Andrey Deviatkin

**Affiliations:** 1 Federal Research Center for Innovator and Emerging Biomedical and Pharmaceutical Technologies, 125315 Moscow, Russia; saiganova_ma@academpharm.ru (M.S.); trutneva-k@mail.ru (K.T.); glazova_ov@academpharm.ru (O.G.); luda4everandever@gmail.com (L.S.); annet_navojkova@mail.ru (A.N.); albert_ea@academpharm.ru (E.A.); mityaeva_on@academpharm.ru (O.M.); volchkov@genlab.llc (P.V.); 2 Federal State Budget Institution of Science Institute of Theoretical and Experimental Biophysics, 142290 Pushchino, Russia; bswin2000@gmail.com; 3 Baikalsky State Nature Biosphere Reserve, 671220 Tankhoi, Russia; janisimov@gmail.com; 4 Department of Fundamental Medicine, Lomonosov Moscow State University, 119992 Moscow, Russia; 5 The Moscow Clinical Scientific Center (MCSC) Named after A.S. Loginov, 111123 Moscow, Russia; 6 Faculty of Bioengineering and Bioinformatics, Lomonosov Moscow State University, 119992 Moscow, Russia

**Keywords:** influenza A virus, reassortment, H13N8, surveillance

## Abstract

Influenza A viruses (IAVs) pose a serious threat to global health. On the one hand, these viruses cause seasonal flu outbreaks in humans. On the other hand, they are a zoonotic infection that has the potential to cause a pandemic. The most important natural reservoir of IAVs are waterfowl. In this study, we investigated the occurrence of IAV in birds in the Republic of Buryatia (region in Russia). In 2020, a total of 3018 fecal samples were collected from wild migratory birds near Lake Baikal. Of these samples, 11 were found to be positive for the H13N8 subtype and whole-genome sequencing was performed on them. All samples contained the same virus with the designation A/Unknown/Buryatia/Arangatui-1/2020. To our knowledge, virus A/Unknown/Buryatia/Arangatui-1/2020 is the first representative of the H13N8 subtype collected on the territory of Russia, the sequence of which is available in the GenBank database. An analysis of reassortments based on the genome sequences of other known viruses has shown that A/Unknown/Buryatia/Arangatui-1/2020 arose as a result of reassortment. In addition, a reassortment most likely occurred several decades ago between the ancestors of the viruses recently collected in China, the Netherlands, the United States and Chile. The presence of such reassortment emphasizes the ongoing evolution of the H13N8 viruses distributed in Europe, North and East Asia, North and South America and Australia. This study underscores the importance of the continued surveillance and research of less-studied influenza subtypes.

## 1. Introduction

Influenza is an enveloped virus with a segmented RNA genome. Influenza viruses are divided into four species: A, B, C and D. Remarkably, influenza A viruses (IAVs) are responsible for most of the major human pandemics, including the Spanish flu of 1918–1920, which killed more than 50 million people [1]. While influenza C and D contain seven genome segments, influenza A and B contain eight. The three largest segments encode viral RNA-dependent RNA polymerases (segments PB1, PB2 and PA), which are responsible for the replication of the viral genome in host cells. Other segments are the nucleoprotein (NP), the matrix protein (M1) and the membrane protein (M2), the nonstructural protein (NS1), the nuclear export protein (NEP) and two segments encoding glycoproteins that envelop the virus, namely haemagglutinin (HA) [1] and neuraminidase (NA) [2]. These two segments play the most important role in the life cycle of the virus, as HA is responsible for binding to cell surface receptors and entering the cell [3], while NA provides the release of new viral particles [2,4]. HA and NA are the most variable antigenically. Therefore, IAV has been categorized into subtypes depending on which HA and NA types are present in the particles. There are 19 different HA and 11 different NA types [5], and each subtype of influenza A has a number depending on which HA and NA types it has, e.g., H1N1 and H3N2 are the subtypes that mainly cause seasonal infections and even pandemics in humans.

Point mutations in HA and NA can accumulate and lead to subtle changes in the surface glycoproteins. This process is called antigenic drift and ensures a smooth evolution of the virus. As a result, the vaccine developed based on the previous year’s strain may be ineffective against the new season’s virus [6]. In contrast, the rapid changes in the viral genome are caused by the so-called antigenic shift of HA or NA. If several different IAV strains infect the same host, the genomes of these strains can reorganize and form a new strain. Antigenic shift and antigenic drift together ensure the evolution of influenza viruses, which complicates the development of vaccines and still makes influenza research relevant today [2,3]. 

IAVs are a group of viruses that circulate in animals, including birds [7,8], pigs [9], bats [10], dogs and cats [11], marine mammals [12] and humans [4]. These viruses cause seasonal epidemics in humans that lead to 300–500 thousand deaths each year [4]. In other words, influenza A viruses can effectively infect different species. However, some subtypes circulate only in one or several species, e.g., the two HA subtypes H13 and H16 occur naturally only in gulls, terns and very rarely in ducks [13]. These subtypes are widespread in the northern hemisphere [14,15] and form two geographically separate clades in Eurasia and North America. These clades are only weakly connected by migratory birds, which only have contact with shorebirds during migration [13]. 

In 2014, Lindsay et al. [16] published the results of a phylogenetic analysis of two H16 samples collected in California, USA (one isolate from 1750 gull feces samples collected in 2012 and one isolate from 300 chickens in 2013). Surprisingly, different genome segments showed 98–99% similarity with samples collected in different years in different regions. The HA, PA, PB1 and PB2 segments were similar to the sequence of the virus collected in Iceland in 2010, the NP segment was similar to the sequence of the virus collected in New Jersey (USA) in 2009, the M sequence was similar to the sequence of the virus collected in Quebec (Canada) in 2009 and the NA sequence was similar to the sequence of the virus collected in Alaska (USA) in 2010. 

Lindh et al. [17] published a study in 2016 based on the analysis of 409 gull samples collected in Finland between 2005 and 2010. Among them, only 14 influenza-positive samples were detected, including 11 H13 samples, 2 H16 and 1 H3N8. Only two HA sequences of H13 were long enough for phylogenetic analysis and showed 98–99% similarity with sequences from viruses collected in Norway, Sweden, Denmark and Central Asia. Another H13 sequence showed 98% identity with North and South American strains. The HA sequences of H16 showed 98% identity with European strains and 98–99% identity with Central Asian strains. 

These and other [18] reports show that H13 and H16 viruses are usually reassortants with a broad geography of ancestors. However, low pathogenicity variants such as H13 and H16 are less studied than highly pathogenic variants because they do not have a major impact on human and domestic bird populations. To investigate the global spread and evolution of H13 and H16 in 2020, Verhagen et al. [19] analyzed the HA sequences of 84 viruses. It was found that H13 and H16 circulate independently in wild birds without overlapping antigenic patterns.

In this study, a complete genome sequence analysis of 122 H13N8 isolates, including samples deposited to the National Center for Biotechnology Information, NCBI, and collected by the authors, is performed to investigate the phylogeography and evolution of low-pathogenic H13 IAVs over a long period of time and on a worldwide geographic scale.

## 2. Materials and Methods

### 2.1. Sample Collection

In September 2020, an expedition was undertaken to collect biological samples in the vicinity of Lake Baikal. The main aim of this expedition was to collect fecal samples from wild migratory birds. A total of 3018 fecal samples were collected. In total, 1986 samples were collected in the delta of the Selenga River in the Republic of Buryatia, and the remaining 1032 samples were collected on the shore of Lake Arangatui, a satellite of Lake Baikal. These particular sampling sites were selected due to their recognized importance as resting areas for birds during their seasonal migrations, with a focus on waterfowl. Waterfowl are known to be important vectors for avian influenza viruses [6].

The fecal samples were collected with cotton swabs to preserve their freshness and integrity. The collected samples were stored in cryotubes to ensure their preservation at low temperatures. To maintain the samples’ viability, all cryotubes were stored in liquid nitrogen dewars for the duration of the expedition. The samples were transported to the laboratory on dry ice. After transport, all samples were stored at −80 °C.

### 2.2. Virus Isolation and Detection

The collected samples (n = 3018) were grouped into 302 pools. Each pool was subjected to RNA isolation followed by real-time PCR analysis using specific primers for the M gene. These primers were recommended by the World Health Organization (WHO) [20]. Of the total 302 sample pools, only five pools were positive in reverse transcription PCR. The samples from these positive pools were then individually subjected to reverse transcription PCR analysis. Further, 50 µg of each positive sample was suspended, dissolved in 500 µL of PBS with 1× gentamicin and mixed well by vortexing. The samples were then centrifuged at 13,000 rpm for 5 min. The supernatant was filtered through a 0.45 μm syringe filter to remove larger particles and potential contaminants. These supernatants were injected into eight-day-old chicken embryos, allowing the viruses to replicate during a four-day incubation period. In other words, live viruses were isolated for tested samples.

The allantoic fluid was extracted from the embryos and collected for further analysis. The collected allantoic fluid was subjected to RNA isolation followed by real-time PCR analysis using primers specific to the M gene.

### 2.3. Virus Sequencing

For further analysis, all positive samples were subjected to one-step RT-PCR (Reverse Transcription Polymerase Chain Reaction) using the SuperScript III One-Step RT-PCR System with Platinum Taq DNA Polymerase Kit, Invitrogen, Carlsbad, CA, USA. In this procedure, we combined both cDNA synthesis (complementary DNA) and PCR amplification in a single tube and used genome-specific primers for all virus fragments: MBTuni-12 [5-ACGCGTGATCAGCAAAAGCAGG] and MBTuni-13 [5-ACGCGTGATCAGTAGTAGAAACAAGG]. Subsequently, the isolated fragments were cloned into the TOPO vector using the TOPO XL-2 Complete PCR Cloning Kit from Invitrogen and then sequenced with M13 primers using the Sanger method. The Sanger reads were assembled with the SnapGene v.2.3.2 software. For segments longer than the Sanger reads, sequencing was performed in several steps. After sequencing with M13 primers, the primers were synthesized for the further sequencing of the remaining part of the segment. 

### 2.4. Preparation of Concatenated Sequences and Alignment

A BLAST search [21] showed that the Baikal sample was representative of the H13N8 subtype. All H13N8 nucleotide sequences of the fragments available from December 2022 were retrieved from the NCBI database [22]. Only full-length sequences were obtained for further analysis. Viral Segment Concatenator [23] was used to concatenate eight viral segment sequences into one artificial sequence. All sequences that differed from another sequence in the dataset by less than 0.1% of the nucleotide sequence were omitted using CD-HIT [24]. The viral sequences were aligned using MAFFT [25] v7.453 (8 November 2019) with the following parameters: mafft --localpair --maxiterate 1000. The Baikal sample (deposited in GenBank under accession numbers OQ868192–OQ868199) was added to the genome pool to perform a reassortment analysis. The sequences of its segments were also concatenated. 

### 2.5. Reassortment Analysis 

Reassortment analysis was conducted using the construction of the pairwise distance correspondence plot (PDCP) and pairwise distance deviation matrix (PDDM) [26]. RDP5 [27] was used to test and compare the results from the general method. SimPlot++ 3.5.1 was used to make similarity plots [28]. Phylogenetic trees were constructed with IQ-TREE [29] using the GTR substitution model and visualized in iTOL [30]. 

## 3. Results

A total of 3018 samples were collected from waterfowl near Lake Baikal. The virus was isolated from the feces of various bird species, so it was not possible to identify the exact host of the virus. At the same time, cormorants and gulls were the predominant bird species in this region in September 2020. A total of 23 out of 3018 samples (0.8%) contained genomic IAV fragments according to the PCR results. In addition, the PCR-positive samples were used for the inoculation of chicken embryos. The collected allantoic fluid was subjected to RNA isolation followed by real-time PCR analysis using primers specific to the M gene. This screening revealed that 11 out of 3018 samples (0.4%) contained viruses that could replicate in chicken embryos. All 11 viruses were subjected to the sequencing.

These sequences of 11 IAVs were completely identical. Since all these samples originate from the same area, we can assume that only one variant of the IAVs is spreading in this bird population (mainly cormorants and gulls). It should be noted that the virus was isolated from the feces of the community of different bird species. Therefore, the identification of the exact host of the virus was not possible. The sequences of the virus segments labelled A/Unknown/Buryatia/Arangatui-1/2020 have been deposited in GenBank under accession numbers OQ868192–OQ868199.

**Figure 1:**
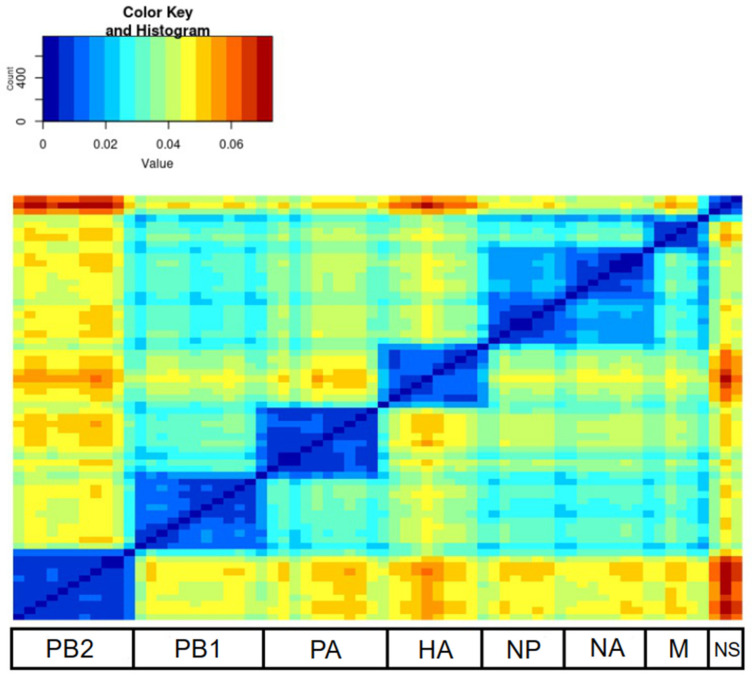
Pairwise distance divergence matrix (PDDM) for H13N8 influenza viruses (Window = 500 nt, Step = 150 bp). The PB2 subunit, hemagglutinin (HA) and NS segments were visually the most involved in recombination events compared to the rest of the genome. The color gradient scale shows the root mean square error (RMSE) values in PDCP.

A total of 976 sequences assigned to the H13N8 subtype were downloaded from GenBank. All segments of 122 viruses were completely sequenced. These segments were sequentially concatenated into a single sequence for each of the 122 H13N8 viruses. The PDDM was generated for all possible pairs of genomic regions using a sliding window (Figure 1). The red in the plot indicates whether there was a high phylogenetic incongruence between the corresponding genomic regions. In contrast, blue indicates a low phylogenetic incongruence between the corresponding genomic regions. Reassortment between different segments was more frequent than in others. The root mean square error (RMSE) of all pairwise distances of two genomic regions from the regression line reflects the extent of phylogenetic incongruence between these fragments of the genome [26]. A high RMSE (shown in red in Figure 1) indicates more frequent reassortment between the corresponding regions of the genome. PDDM showed that reassortment between most divergent viruses occurred between the PB2, HA and NS segments (Figure 1).

**Figure 2:**
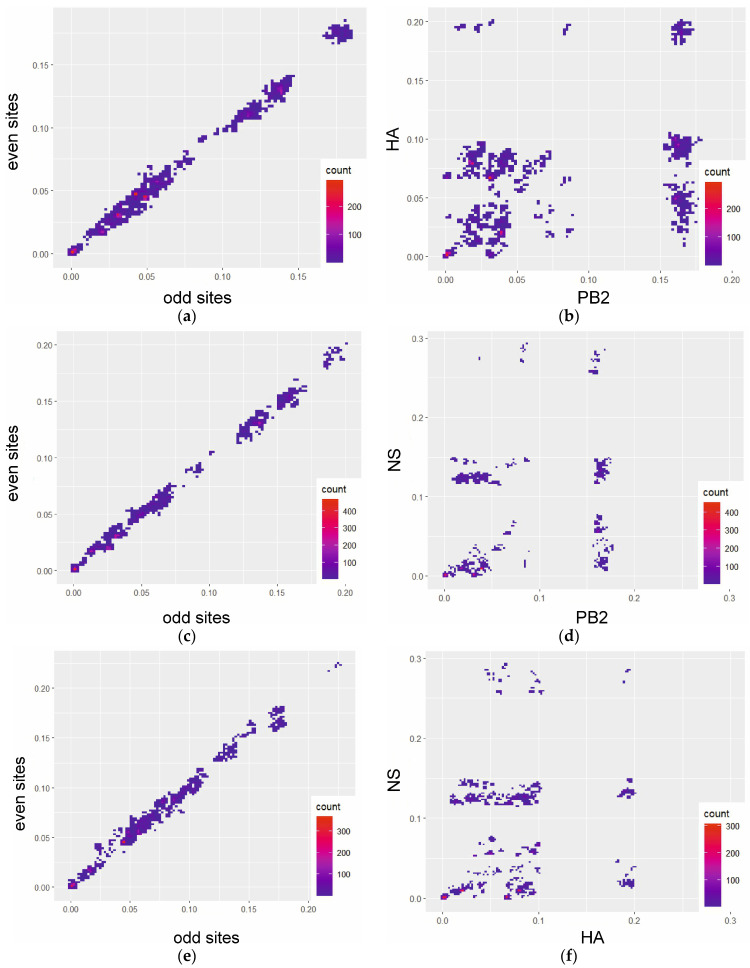
Pairwise nucleotide distance comparison plots (PDCP) showing phylogenetic incongruence between selected genetic regions of H13N8 influenza viruses. Each dot represents a pair of raw nucleotide distances between the two sequences in two genomic regions (axis labelling). (**a**) PDCP constructed for the concatenated 4th (HA) and 8th (NA) segments for which pairwise distances were calculated for even and odd sites (negative control); (**b**) PDCP constructed for HA and NS segments of H13N8 influenza viruses; (**c**) PDCP constructed for even and odd sites of concatenated 2nd (PB2) and 8th (NA) segments (negative control); (**d**) PDCP constructed for PB2 and NS segments of H13N8 influenza viruses; (**e**) PDCP constructed for even and odd sites of concatenated 4th (HA) and 8th (NS) segments (negative control); (**f**) PDCP constructed for HA and NS segments of H13N8 influenza viruses.

PDCPs were generated for the PB2, HA and NS segments to show the difference in the pairwise evolutionary distances between these genomic regions (Figure 2). Briefly, the percentage of different nucleotides between the PB2 and HA segments (Figure 2b), PB2 and NS segments (Figure 2d), HA and NS segments (Figure 2f) were plotted. For the PDCP construction, all sequences were divided into all possible pairs. In the next step, the percentage of the different nucleotides for the different genomic regions was calculated (axes in Figure 2), which determined the position of the dots in the PDCP. It should be noted that virus pairs with different percentages of different nucleotides in different genomic regions are assumed to contain reassortant viruses. For example, the dots in the lower right area of Figure 2f indicate the virus pairs that differ by about 18% of the nucleotides in the PB2 segment sequences, but are almost identical in the NS segment sequences. As a negative control for reassortment, the identified genomic regions were concatenated and pairwise evolutionary distances were calculated for the odd and even positions of the same sequence pairs (Figure 2a,c,e). 

**Figure 3:**
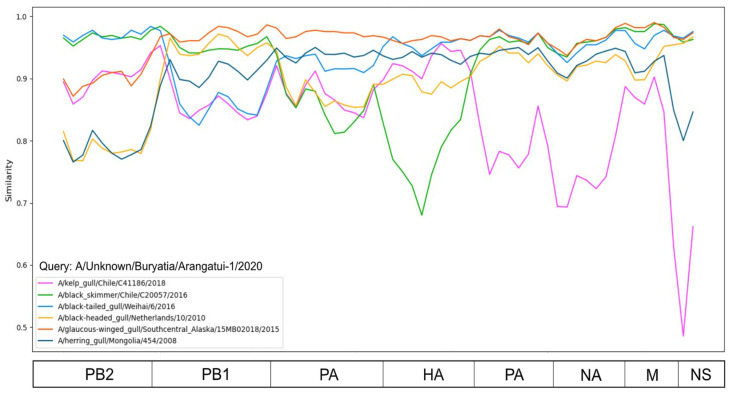
Similarity plot analysis (window = 500, step = 200) based on different segments of Baikal sample. The *x*-axis shows the segments, and the *y*-axis shows the percent similarity between the query sequence and six other chosen viruses. The genome of A/Unknown/Buryatia/Arangatui-1/2020 was selected as the query sequence.

**Figure 4:**
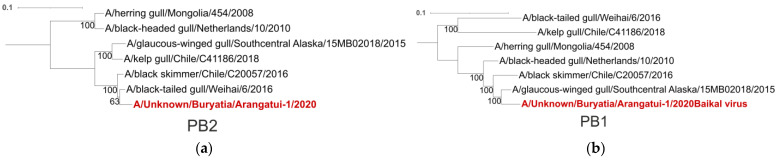

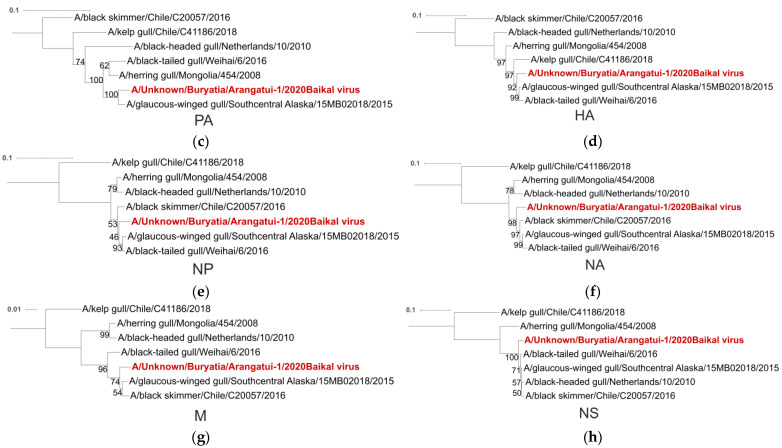
Phylogenetic trees were constructed for each segment, namely PB2 (**a**), PB1 (**b**), PA (**c**), HA (**d**), NP (**e**), NA (**f**), M (**g**) and NS (**h**), of the influenza A genome. A/Unknown/Buryatia/Arangatui-1/2020 sample is highlighted in red. Maximum likelihood (ML) phylogenetic inference was inferred from the MAFFT alignment [31] using IQ-TREE. The reliability of the trees was evaluated by UFBoot values [25]. The scale bar indicates the nucleotide substitutions per site.

Similarity plot analysis (Figure 3) was performed for the virus collected near Lake Baikal and six other viruses that formed virus pairs with the virus we collected, which had different numbers of non-identical nucleotides in different genomic regions, as indicated by the PDCP (Figure 2). The intersections of the similarity plots were taken as an indication of multiple reassortments. To further validate the results, phylogenetic trees were generated for each segment. The different topology of these trees also indicates reassortment events (Figure 4). According to the phylogenetic trees (Figure 4), each segment of A/Unknown/Buryatia/Arangatui-1/2020 was grouped with different viruses isolated at remote locations (Table 1). For the PB2 segment, the most similar viruses were isolated from China and Chile; for PB1 and PA, from the USA; for HA, from the USA and China; for NP and NA, from the USA, China and Chile; for M, from the USA and Chile; and for NS, from the USA, China, Chile and the Netherlands. It should be noted that here the grouping of the virus with China A/Unknown/Buryatia/Arangatui-1/2020 was determined as belonging to those clades of the phylogenetic tree that contained novel viruses and had a bootstrap support value of more than 70 at the corresponding node.

## 4. Discussion

Reassortment in IAVs can occur either between viruses of the same subtype or between viruses of different subtypes. Influenza viruses isolated from migratory birds can have a diverse genetic constellation with a combination of internal genes from different lineages or variants. For example, the similarity plot (Figure 3) and phylogenetic trees (Figure 4, Table 1) show the mosaic nature of the genome of virus A/Unknown/Buryatia/Arangatui-1/2020. A comparison with viruses of the same subtype (H13N8) shows that the genetic diversity of segments of the selected viruses can change drastically. For example, the HA segment of A/Unknown/Buryatia/Arangatui-1/2020 and A/Black skimmer/Chile/C20057/2016 differ in 316 out of 1660 nucleotides (19.0%) according to the BLASTN algorithm, while the PB2 segment sequences of these two viruses are not identical in 72 out of 2279 (3.2%) nucleotides according to the BLASTN algorithm. The most plausible explanation for this observation is that the ancestor of virus A/Unknown/Buryatia/Arangatui-1/2020 or A/Black skimmer/Chile/C20057/2016 lost its original HA segment and gained a new HA segment through a reassortment event.

The most plausible explanation for the contradictory topology of the phylogenetic trees constructed for the different segments of the selected H13N8 viruses (Figure 4, Table 1) is an abundant process of reassortment that generally occurs in H13N8 [32]. At the same time, it should be noted that one of the most important factors for the global diversity of H13 viruses is intersubtypic reassortment. For example, A/Vega gull/South Korea/GNU54/2021(H13N6), which is most similar in sequence to the PB2 segment of A/Unknown/Buryatia/Arangatui-1/2020, is a representative of a different subtype. At the same time, the PB2 segment sequences of these two viruses match 2259 of 2279 nucleotides (99.12%). The closest representatives of H13N8, A/black-tailed gull/Weihai/17/2016 and A/black skimmer/Chile/C20057/2016, have nucleotides that are 97.19% and 96.75% identical to the PB2 segment sequence of A/Unknown/Buryatia/Arangatui-1/2020, respectively. The mean evolutionary rate of H13 was estimated to be approximately 5.81 × 10^−3^ substitutions per site per year [33]. This means that about 0.0581% of the nucleotides of the H13 genome change every year. In other words, according to a rough estimate, the most recent common ancestor (tMRCA) of the PB2 segment of A/Unknown/Buryatia/Arangatui-1/2020 and A/Vega gull/South Korea/GNU54/2021 existed about ten years ago, while the tMRCA of A/Unknown/Buryatia/Arangatui-1/2020 and A/black skimmer/Chile/C20057/2016, for example, existed decades ago. At the same time, according to the NA segment sequences available in the GeneBank database, A/yellow-legged gull/Republic of Georgia/1/2013, A/black-headed gull/Netherlands/7/2013, A/black skimmer/Chile/C20057/2016, A/black-tailed gull/Weihai/17/2016 and A/Glaucus-winged gull/Southcentral Alaska/15MB02018/2015 show nucleotides that are 95.19% to 97.24% identical to A/Unknown/Buryatia/Arangatui-1/2020, indicating that the tMRCA of the above viruses NA segments has not existed recently. 

In other words, the genome of strain A/Unknown/Buryatia/Arangatui-1/2020 contains traces of reassortments that took place several decades ago between the ancestors of viruses recently collected in China, the Netherlands, the United States and Chile. This means that co-infection of the waterfowl with viruses from Eurasian and North American lineages has occurred in the past. Indeed, migratory birds can fly between North America and Europe, for example, via the East Atlantic Flyway [15], creating a common network of the H13N8 IAV gene pool that spreads across the Americas and Eurasia. This speculation is consistent with the fact that the intensive intercontinental exchange of H13 viruses has recently been demonstrated [19]. It should be noted that, for example, there were none nearly identical to the A/Unknown/Buryatia/Arangatui-1/2020 sequences of H13N8 viruses. The closest known viral sequences in terms of the percentage of identical nucleotides showed considerable differences in their genomes. This means that the description of the genome sequence of the virus collected at Lake Baikal significantly expands our knowledge about the genetic diversity of H13N8. On the other hand, it shows that knowledge about the actual diversity of this low-pathogenic IAV subtype is still very fragmentary. At the same time, H13N8 viruses spread with waterfowl flying in northern Eurasia and North and South America, and currently there is no standardized system of global surveillance. 

Recently, it has been shown that H13 representatives, A/mallard/Dalian/DZ-137/2013 and A/Eurasian Curlew/Liaoning/ZH-385/2014 may infect chickens and mice [34]. This means that H13 viruses may infect not only wild aquatic birds but also domesticated birds and mammals. Indeed, serological analysis by hemagglutination inhibition demonstrated the recent infection of birds housed in farms (e.g., muscovy ducks, geese, swan geese, mallards, pheasants, red-legged partridges, rock partridges, grey partridges). Moreover, antibodies against H13 IAV were found in humans working as wildlife professionals in Italy [35]. At the same time, to the best of our knowledge, currently there are no known sequences of H13 viruses collected from humans. 

Logically, most of the resources are used to research those variants of the influenza virus that pose an immediate threat to humans and have pandemic potential. In February 2024, for example, GenBank contained 153,470 and 155,681 records belonging to the H1N1 and H2N3 subtypes of IAVs that cause seasonal outbreaks of this infection in humans. Of particular interest are the highly pathogenic variants of the influenza virus, which are widespread among birds and can cause fatal infections in humans. For example, serotypes H5N1 and H5N8 are considered highly pathogenic and are the subject of increased interest. In February 2024, the GenBank database contained 30,856 and 8596 data records for these variants. Of note, reassortment is one of the most important drivers of IAV evolution. Highly pathogenic variants can arise through reassortment with low-pathogenic viruses. For example, the highly pathogenic H5N6 avian influenza virus emerged through reassortment between the highly virulent H5N8 strain from Korea and the N6 gene of a low pathogenic H3N6 virus from the Netherlands [36]. Thus, even low-pathogenic variants of the influenza virus can contribute to the emergence of new highly pathogenic variants and pose a serious threat to global health. For example, the spread of highly pathogenic H5 IAVs mostly overlaps with the distribution of H13N8 IAVs [37]. For this reason, co-infection of the same bird with two different viruses is possible, probably leading to the emergence of a new variant of the virus with unpredictable phenotypic characteristics.

It should be noted that 1173 sequences for the low-pathogenic variant H13N8 are currently described in the GenBank database (as of February 2024). The monitoring of the spread of the different influenza virus serotypes is therefore inconsistent. In addition, the intensity of the detection of the genomic sequences of viruses of the same subtype varies greatly in different regions of the world. To our knowledge, the A/Unknown/Buryatia/Arangatui-1/2020 virus is the first representative of subtype H13N8 collected on the territory of Russia, the sequence of which is available in the GenBank database. Of the 1173 entries in GenBank, 717 contain information on viruses collected in the Netherlands, 250 contain information on viruses collected in the USA (including 218 in the state of Minnesota), 80 contain information on viruses collected in the Republic of Georgia, 56 contain information on viruses collected in Chile, 40 contain information on viruses collected in China, 19 contain information on viruses collected in Sweden, 8 contain information on virus collected in Mongolia, 2 contain information on viruses collected in Australia and 1 contains information on virus collected in Norway. 

A quick analysis of the strain names of the H13N8 subtype listed in the GenBank database thus shows that its actual distribution is significantly higher than described in earlier studies. In addition to North America and Eurasia, these viruses have also been found in South America and Australia. For example, H13N8 representatives have been found in the US states of Minnesota and Alaska. However, viruses have yet to be discovered and collected in Canada, which lies between these two locations. No viruses have been described in the region between China and Australia either. In Europe, the virus has only been found in the Netherlands, Sweden and Norway. At the same time, antibodies against H13N8 have been found in poultry in Italy. Overall, this analysis indicates a wider and possibly underestimated distribution of the H13N8 subtype, with the GenBank database providing valuable information on the presence of the virus in different geographical regions. The discrepancies in distribution emphasize the need for a continued global surveillance system for IAVs transmitted by migratory birds.

## 5. Conclusions

This study presents a comprehensive analysis of H13N8 influenza viruses, focusing on the prevalence of reassortment events within this particular subtype. The research provides valuable insights into the global distribution and evolution of H13N8 influenza viruses, which are classified as a low-pathogenic subtype found in wild birds. By analyzing 122 full-genome sequences, this study reveals the widespread reassortment events occurring within the H13N8 subtype. It should be noted that the segment exchange between most divergent viruses occurred between the PB2, HA and NS segments.

The identification of a new reassortant virus near Lake Baikal highlights the critical need for continuous surveillance and research to better understand the evolutionary dynamics of influenza viruses, particularly in less studied subtypes such as H13N8.

The limited availability of complete genomes in public databases underscores the need for further investigations and sequencing efforts to enhance our understanding of the genetic diversity and evolution of H13N8 and other less-studied influenza subtypes.

## Figures and Tables

**Table 1:** Viruses clustering with A/Unknown/Buryatia/Arangatui-1/2020 at high bootstrap support (according to Figure 4).

Segment	Closest Viruses	Place of Collection
PB2	A/Black-tailed gull/Weihai/6/2016	China
	A/Black skimmer/Chile/C20057/2016	Chile
PB1	A/Glaucus-winged gull/Southcentral Alaska/15MB02018/2015	USA
PA	A/Glaucus-winged gull/Southcentral Alaska/15MB02018/2015	USA
HA	A/Glaucus-winged gull/Southcentral Alaska/15MB02018/2015	USA
	A/Black-tailed gull/Weihai/6/2016	China
NP	A/Glaucus-winged gull/Southcentral Alaska/15MB02018/2015	USA
	A/Black-tailed gull/Weihai/6/2016	China
	A/Black skimmer/Chile/C20057/2016	Chile
NA	A/Glaucus-winged gull/Southcentral Alaska/15MB02018/2015	USA
	A/Black-tailed gull/Weihai/6/2016	China
	A/Black skimmer/Chile/C20057/2016	Chile
M	A/Glaucus-winged gull/Southcentral Alaska/15MB02018/2015	USA
	A/Black skimmer/Chile/C20057/2016	Chile
NS	A/Glaucus-winged gull/Southcentral Alaska/15MB02018/2015	USA
	A/Black-tailed gull/Weihai/6/2016	China
	A/Black skimmer/Chile/C20057/2016	Chile
	A/Black-headed gull/Netherlands/10/2010	Netherlands

## Data Availability

The alignment file of the concatenated H13N8 virus genomes in FASTA format is available at https://github.com/AndreiDeviatkin/repo/blob/main/h13n8.fasta (accessed on 5 April 2024).

## References

[1] Martini M., Gazzaniga V., Bragazzi N.L., Barberis I. (2019). The Spanish Influenza Pandemic: A Lesson from History 100 Years after 1918. J. Prev. Med. Hyg..

[2] Creytens S., Pascha M.N., Ballegeer M., Saelens X., de Haan C.A.M. (2021). Influenza Neuraminidase Characteristics and Potential as a Vaccine Target. Front. Immunol..

[3] Wu N.C., Wilson I.A. (2020). Influenza Hemagglutinin Structures and Antibody Recognition. Cold Spring Harb. Perspect. Med..

[4] Krammer F., Smith G.J.D., Fouchier R.A.M., Peiris M., Kedzierska K., Doherty P.C., Palese P., Shaw M.L., Treanor J., Webster R.G. (2018). Influenza. Nat. Rev. Dis. Prim..

[5] Fereidouni S., Starick E., Karamendin K., Di Genova C., Scott S.D., Khan Y., Harder T., Kydyrmanov A. (2023). Genetic Characterization of a New Candidate Hemagglutinin Subtype of Influenza A Viruses. Emerg. Microbes Infect..

[6] Webster R.G., Bean W.J., Gorman O.T., Chambers T.M., Kawaoka Y. (1992). Evolution and Ecology of Influenza A Viruses. Microbiol. Rev..

[7] Li Y.-T., Linster M., Mendenhall I.H., Su Y.C.F., Smith G.J.D. (2019). Avian Influenza Viruses in Humans: Lessons from Past Outbreaks. Br. Med. Bull..

[8] Blagodatski A., Trutneva K., Glazova O., Mityaeva O., Shevkova L., Kegeles E., Onyanov N., Fede K., Maznina A., Khavina E. (2021). Avian Influenza in Wild Birds and Poultry: Dissemination Pathways, Monitoring Methods, and Virus Ecology. Pathogens.

[9] Crisci E., Mussá T., Fraile L., Montoya M. (2013). Review: Influenza Virus in Pigs. Mol. Immunol..

[10] Campos A.C.A., Góes L.G.B., Moreira-Soto A., de Carvalho C., Ambar G., Sander A.-L., Fischer C., Ruckert da Rosa A., Cardoso de Oliveira D., Kataoka A.P.G. (2019). Bat Influenza A(HL18NL11) Virus in Fruit Bats, Brazil. Emerg. Infect. Dis..

[11] Borland S., Gracieux P., Jones M., Mallet F., Yugueros-Marcos J. (2020). Influenza A Virus Infection in Cats and Dogs: A Literature Review in the Light of the “One Health” Concept. Front. Public Health.

[12] Runstadler J.A., Puryear W. (2020). A Brief Introduction to Influenza A Virus in Marine Mammals. Methods Mol. Biol..

[13] Hall J.S., TeSlaa J.L., Nashold S.W., Halpin R.A., Stockwell T., Wentworth D.E., Dugan V., Ip H.S. (2013). Evolution of a Reassortant North American Gull Influenza Virus Lineage: Drift, Shift and Stability. Virol. J..

[14] Wille M., Robertson G.J., Whitney H., Bishop M.A., Runstadler J.A., Lang A.S. (2011). Extensive Geographic Mosaicism in Avian Influenza Viruses from Gulls in the Northern Hemisphere. PLoS ONE.

[15] Dusek R.J., Hallgrimsson G.T., Ip H.S., Jónsson J.E., Sreevatsan S., Nashold S.W., TeSlaa J.L., Enomoto S., Halpin R.A., Lin X. (2014). North Atlantic Migratory Bird Flyways Provide Routes for Intercontinental Movement of Avian Influenza Viruses. PLoS ONE.

[16] Lindsay L.L., Plancarte M., Brenn-White M., Boyce W.M. (2014). Complete Genome Sequences of the First Reported California H16 Influenza A Viruses. Genome Announc..

[17] Lindh E., Ek-Kommonen C., Isomursu M., Alasaari J., Vaheri A., Vapalahti O., Huovilainen A. (2017). Genetic characterization of H13 and h16 influenza a viruses in gulls (*Larus* spp.) with clinically severe disease and concurrent circovirus infection. J. Wildl. Dis..

[18] Yu Z., He H., Cheng K., Wu J., Gao Y., Chen W., Yuan X., Zhao Y. (2019). Genetic Characterization of an H13N2 Low Pathogenic Avian Influenza Virus Isolated from Gulls in China. Transbound. Emerg. Dis..

[19] Verhagen J.H., Poen M., Stallknecht D.E., van der Vliet S., Lexmond P., Sreevatsan S., Poulson R.L., Fouchier R.A.M., Lebarbenchon C. (2020). Phylogeography and Antigenic Diversity of Low-Pathogenic Avian Influenza H13 and H16 Viruses. J. Virol..

[20] WHO Influenza Virus Detection Protocols. https://cdn.who.int/media/docs/default-source/influenza/molecular-detention-of-influenza-viruses/protocols_influenza_virus_detection_feb_2021.pdf?sfvrsn=df7d268a_5.

[21] Johnson M., Zaretskaya I., Raytselis Y., Merezhuk Y., McGinnis S., Madden T.L. (2008). NCBI BLAST: A Better Web Interface. Nucleic Acids Res..

[22] Clark K., Karsch-Mizrachi I., Lipman D.J., Ostell J., Sayers E.W. (2016). GenBank. Nucleic Acids Res..

[23] Ivanova A.O., Volchkov P.Y., Deviatkin A.A. (2024). Concatenation of Segmented Viral Genomes for Reassortment Analysis. bioRxiv.

[24] Fu L., Niu B., Zhu Z., Wu S., Li W. (2012). CD-HIT: Accelerated for Clustering the next-Generation Sequencing Data. Bioinformatics.

[25] Katoh K., Standley D.M. (2013). MAFFT Multiple Sequence Alignment Software Version 7: Improvements in Performance and Usability. Mol. Biol. Evol..

[26] Vakulenko Y., Deviatkin A., Drexler J.F., Lukashev A. (2021). Modular Evolution of Coronavirus Genomes. Viruses.

[27] Martin D.P., Varsani A., Roumagnac P., Botha G., Maslamoney S., Schwab T., Kelz Z., Kumar V., Murrell B. (2021). RDP5: A Computer Program for Analyzing Recombination in, and Removing Signals of Recombination from, Nucleotide Sequence Datasets. Virus Evol..

[28] Samson S., Lord É., Makarenkov V. (2022). SimPlot++: A Python Application for Representing Sequence Similarity and Detecting Recombination. Bioinformatics.

[29] Minh B.Q., Schmidt H.A., Chernomor O., Schrempf D., Woodhams M.D., von Haeseler A., Lanfear R. (2020). Corrigendum to: IQ-TREE 2: New Models and Efficient Methods for Phylogenetic Inference in the Genomic Era. Mol. Biol. Evol..

[30] Letunic I., Bork P. (2021). Interactive Tree of Life (ITOL) v5: An Online Tool for Phylogenetic Tree Display and Annotation. Nucleic Acids Res..

[31] Katoh K., Rozewicki J., Yamada K.D. (2018). MAFFT Online Service: Multiple Sequence Alignment, Interactive Sequence Choice and Visualization. Brief. Bioinform..

[32] Meng B., Wang Q., Leng H., Ren C., Feng C., Guo W., Feng Y., Zhang Y. (2024). Evolutionary Events Promoted Polymerase Activity of H13N8 Avian Influenza Virus. Viruses.

[33] Hill N.J., Bishop M.A., Trovão N.S., Ineson K.M., Schaefer A.L., Puryear W.B., Zhou K., Foss A.D., Clark D.E., MacKenzie K.G. (2022). Ecological Divergence of Wild Birds Drives Avian Influenza Spillover and Global Spread. PLOS Pathog..

[34] Sun W., Zhao M., Yu Z., Li Y., Zhang X., Feng N., Wang T., Wang H., He H., Zhao Y. (2023). Cross-Species Infection Potential of Avian Influenza H13 Viruses Isolated from Wild Aquatic Birds to Poultry and Mammals. Emerg. Microbes Infect..

[35] De Marco M.A., Delogu M., Facchini M., Di Trani L., Boni A., Cotti C., Graziosi G., Venturini D., Regazzi D., Ravaioli V. (2021). Serologic Evidence of Occupational Exposure to Avian Influenza Viruses at the Wildfowl/Poultry/Human Interface. Microorganisms.

[36] Kim Y.I., Si Y.J., Kwon H.I., Kim E.H., Park S.J., Robles N.J., Nguyen H.D., Yu M.A., Yu K.M., Lee Y.J. (2018). Pathogenicity and Genetic Characterisation of a Novel Reassortant, Highly Pathogenic Avian Influenza (HPAI) H5N6 Virus Isolated in Korea, 2017. Eurosurveillance.

[37] Huang P., Sun L., Li J., Wu Q., Rezaei N., Jiang S., Pan C. (2023). Potential Cross-Species Transmission of Highly Pathogenic Avian Influenza H5 Subtype (HPAI H5) Viruses to Humans Calls for the Development of H5-Specific and Universal Influenza Vaccines. Cell Discov..

